# Preparation of Superhydrophobic Film on Ti Substrate and Its Anticorrosion Property

**DOI:** 10.3390/ma10060628

**Published:** 2017-06-08

**Authors:** Min Zhu, Wenchuan Tang, Luyao Huang, Dawei Zhang, Cuiwei Du, Gaohong Yu, Ming Chen, Thee Chowwanonthapunya

**Affiliations:** 1 Corrosion and Protection Center, Institute for Advanced Materials and Technology, University of Science and Technology Beijing, Beijing 100083, China; wenchuantang99@126.com (W.T.); luyaohuang99@126.com (L.H.); dcw@ustb.edu.cn (C.D.); 2 School of Mechanical Engineering & Automation, Zhejiang Sci-Tech University, Hangzhou 310018, China; zmii2009@163.com (M.Z.); ghyuzstu@163.com (G.Y.); mingchenzstu@126.com (M.C.); 3 Faculty of International Maritime Studies, Kasetsart University, Sriracha Campus, 199 Tungsukla, Sriracha, Chonburi 20230, Thailand

**Keywords:** superhydrophobic film, titanium, microstructure, surface

## Abstract

Superhydrophobic films were fabricated on a titanium substrate with or without anodizing by using a self-assembling method. Firstly, the pretreatments of mechanical polishing/anodizing or mechanical polishing only were conducted, respectively. Subsequently, the preparation of polydopamine film layer, deposition of nano-silver particles, and post modification of 1H,1H,2H,2H-perfluorodecanethiol were performed on the surface of the pretreated substrate. The surface morphologies, compositions, wettability, and corrosion resistance of the films were investigated with scanning electron microscopy (SEM), energy-dispersive spectrometry (EDS), water contact angle measurements, and electrochemical tests, respectively. Meanwhile, the effect of the deposition time in the silver nitrate solution on the hydrophobicity of the specimen surface was investigated. The result showed that with the increase of deposition time, the hydrophobic property enhanced gradually. The surface deposited for 7 h exhibited an optimum hydrophobic effect, which was characterized with a large water contact angle (WCA) of 154°, and the surface was rather rough and covered by a relatively uniform layer of micro-nano silver particles. The excellent hydrophobicity was attributed to a rough stratified microstructure along with the low surface energy. The electrochemical measurements showed that the existence of the superhydrophobic film can effectively enhance the corrosion resistance of Ti samples.

## 1. Introduction

Preparation of super-hydrophobic surfaces, with water contact angles larger than 150°, is an active area of research in view of both fundamental studies and practical applications [1,2,3]. In recent years, superhydrophobic surfaces have received widespread attention because of their non-wetting and self-cleaning surface properties. In nature, a lot of biological surfaces [4,5], such as lotus leaves, exhibit high superhydrophobic and self-cleaning functions, which is known as the “lotus effect”. Conventionally, superhydrophobicity is the result of a combination of hierarchical surface roughness with low surface energy [6]. The mechanisms of superhydrophobicity were explained by the Cassie–Baxter model [7] and Wenzel’s model [8]. Until now, plentiful methods have been developed to fabricate the rough hydrophobicity surfaces, including chemical etching [9], templating [10], anodic oxidization [11], electrodepostion [12,13], and sol–gel processes [14]. However, most methods for fabricating superhydrophobic surfaces usually involve complex processes, expensive equipment, and rigorous preparation conditions. Due to these shortcomings, the above-mentioned methods were limited. Therefore, it is much more attractive to use a simple methodology.

Recently, fabrication of superhydrophobic film has emerged as a new and effective approach to improve the corrosion resistance of metals [15]. The film as the physical barrier could decrease water wetting, and prevent the corrosive medium from contacting with the metal substrates, thereby enhancing their corrosion resistance. Superhydrophobic surfaces have been successfully constructed on various metallic materials [16,17,18,19,20]. To date, there are many reports on the fabrication of superhydrophobic Ti surfaces by various methods [21,22,23,24]. Similarly, the further applications of this technique might be limited due to the complex treatment procedures. In this work, a simplified method without an anodizing step was developed to fabricate superhydrophobic surfaces on Ti substrates. It involved mechanical polishing, and subsequent self-assemblies of dopamine and nano-silver particles. Finally, the surface was further modified in 1H,1H,2H,2H-perfluorodecanethiol. In addition, the corrosion resistance of the Ti samples with or without superhydrophobic films were measured by potentiodynamic polarization curves and electrochemical impedance spectroscopy (EIS) in a 3.5 wt % NaCl solution.

## 2. Experimental

Pure titanium sheets (Ti 99.8 wt %) were cut into small wafers with a diameter of 15 mm (1 mm in thickness). The Ti specimens were used as anodes, and a platinum plate as the cathode, for anodization. Prior to the anodizing process, Ti samples were firstly abraded using 1500 grit SiC sandpaper, followed by ultrasonic cleaning in acetone, alcohol, and deionized water for 15 min, respectively, and finally blow-dried with warm air. Then the Ti specimens were activated by immersing in a 0.5 wt % NH_4_F aqueous solution of ethylene glycol and water with a volume ratio of 99:1 for 5 h at the room temperature (about 15 °C). A constant DC voltage of 20 V for anodization was applied to the electrodes using a high-precision DC power supply. After anodizing, the specimens were washed thoroughly with deionized water and subsequently dried. 

A polydopamine film (PDA) layer was constructed on the surface of the mechanically polished and anodized Ti specimen by directly immersing into the mixed solution for 24 h at room temperature under stirring. The solution (pH = 8.5, adjusted by HCl) was prepared by dissolving 0.12 g tris and 200 mg dopamine hydrochloride in 100 mL water. Subsequently, the nano-silver particles (AgNPs) were deposited on the PDA film by immersing the sample in the silver nitrate solution (1 mg/mL) for 7 h without exposure to light. After deposition, the samples were rinsed with ethanol and dried in an oven at 30 °C. By the self-assemblies of PDA and AgNPs, special stratified microstructures were formed on the Ti substrate. After that, the specimens were modified in a sealed anaerobic ethanol solution of 1H,1H,2H,2H-perfluorodecanethiol with a volume ratio of 500:1 for 12 h at room temperature. Finally, the specimens were dried at 60 °C for 1 h. Similarly, superhydrophobic surfaces were prepared without the anodizing process for comparison of surface morphology and hydrophobic property. Meanwhile, the influence of deposition time (0.5 h, 2 h, 5 h, 7 h, 12 h) of AgNPs on the hydrophobicity of the super-hydrophobic surface was investigated. 

The surface morphology and chemical composition of the surfaces were investigated using scanning electron microscopy (SEM, Quanta 250F, FEI, Hillsboro, OR, USA) and energy-dispersive spectrometry (EDS, OxfordAZtech x-max 80, Oxford Instruments, Oxfordshire, UK). The water contact angle (WCA) was measured by a JC2000A contact angle instrument (Shanghai Zhongchen Digital Technology Apparatus Co., Ltd, Shanghai, China) at ambient temperature. A 5 μL water drop was used. The WCA value was obtained by averaging the measured data at five randomly-selected points.

Electrochemical measurements were performed via a PARSTAT 2273 electrochemical workstation in a three-electrode cell system, where the Ti specimens with or without the superhydrophobic film were used as the working electrode, a platinum plate as the counter electrode, and a saturated calomel electrode (SCE) as the reference electrode. The tests were carried out at ambient temperature in a 3.5 wt % NaCl solution (pH 8.02). Prior to testing, the specimen was maintained for 0.5 h in the solution to ensure that a steady state value of corrosion potential was reached. EIS testing was conducted at the open circuit potential over a frequency range from 100 kHz to 10 mHz with the applied 10 mV signal amplitude. Potentiodynamic polarization curves were measured from −0.250 V (vs. OCP) to 1.850 V (vs. OCP) at a potential sweeping rate of 0.333 mV/s. The exposed area of the working electrode was 1 cm^2^.

## 3. Results and Discussion

### 3.1. Superhydrophobic Film Fabricated by Anodizing

**Figure 1:**
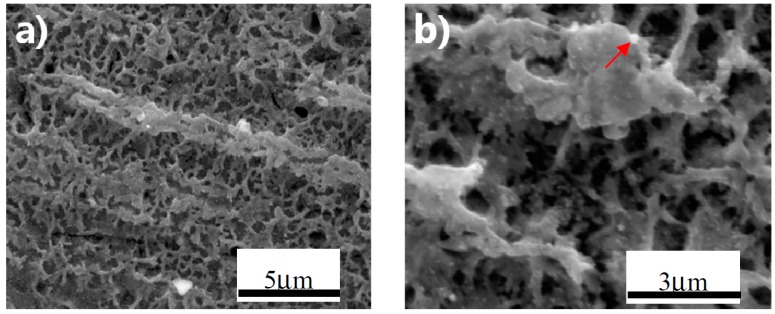
Microstructure of the superhydrophobic surface of an anodized specimen at different magnifications: (**a**) 15,000×; and (**b**) 30,000×.

**Figure 2:**
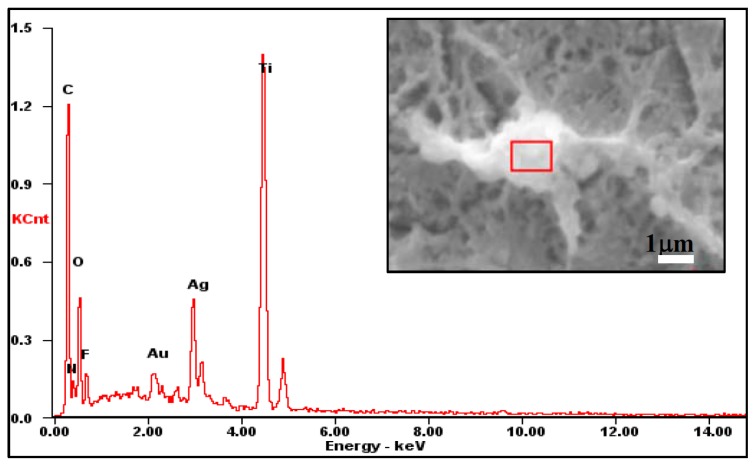
EDS analysis of the anodized superhydrophobic surface.

In Figure 1, the surface morphology of the anodized superhydrophobic film was characterized with a special morphological fragmented-landform structure, which was possibly formed by the collapsed TiO_2_ nanotubes from anodization [25]. Figure 2 exhibits the EDS result of the small bright particle on the superhydrophobic surface. Further amplification of the surface reveals small bright particles distributed throughout the entire surface (Figure 1b, indicated with red arrows), which may be nano-silver particles (AgNPs) formed by the chelation and reduction of Ag ions with the polydopamine. As shown in Figure 2 and Table 1, the existence of Ag was also confirmed by the EDS measurement.

**Figure 3:**
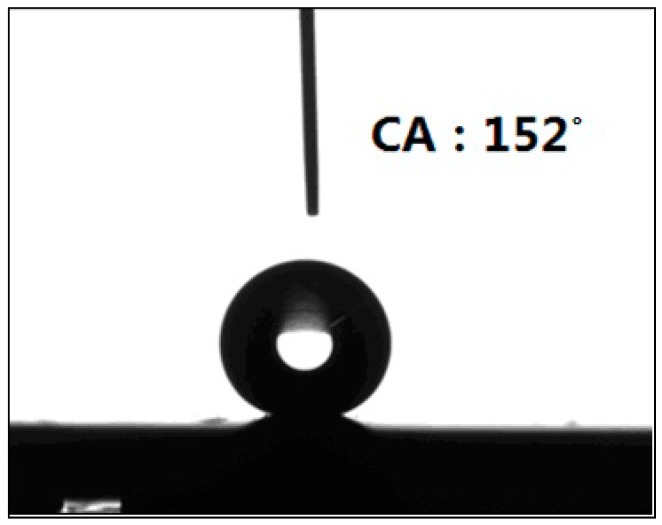
Water contact angle measured for superhydrophobic surface of an anodized sample.

Above all, with the combination of anodizing method and the self-assemblies of dopamine and AgNPs, a multilayer micro-nano structure on the titanium surface was successfully prepared. Through the connection of AgNPs and thiol bonds in the perfluorinated decanethiol hydrophobic segments, a superhydrophobic film was finally constructed. Figure 3 displays that the static WCA on the surface of this film reached ~152° and the sliding angle was less than 5°, demonstrating that a superhydrophobic state was achieved.

### 3.2. Superhydrophobic Film Fabricated without Anodic Oxidation

**Figure 4:**
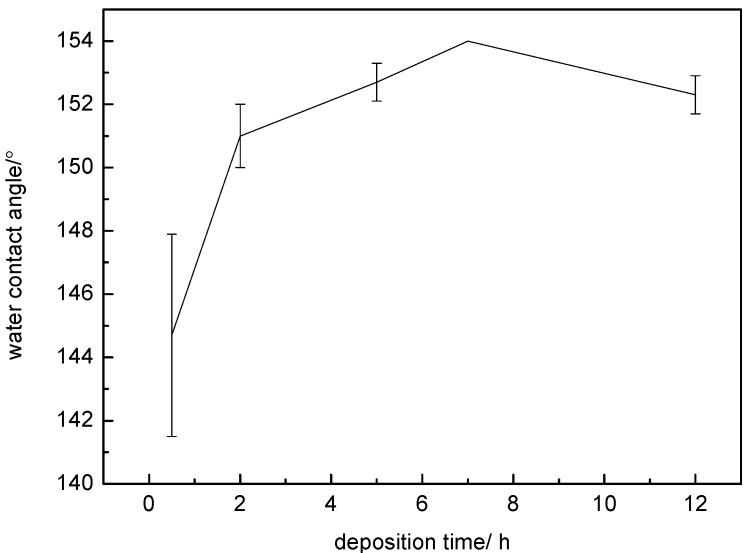
Influence of the deposition time on the hydrophobicity of the superhydrophobic surface.

During the anodic oxidation process, the surface roughness can be influenced by many factors [25], and the preparation process is relatively complex. In order to simplify and control the sample fabrication well, the procedure of anodic oxidation was removed, i.e., only mechanical polishing (1500#) as the pretreatment step was adopted to fabricate the superhydrophobic surface. The results are as follows:

Figure 4 shows the influence of deposition time on the hydrophobicity of the as-prepared surface. The specimen surfaces treated with silver nitrate solution at various deposition times exhibited different WCAs. The WCAs were listed in Table 2. When the deposition time was very short (0.5 h), the surface had a relatively low average WCA of ~145°. With the increasing deposition time, the hydrophobic property was enhanced significantly. The WCA of this surface deposited for 2 h increased to ~151°. By further increasing the time to 7 h, the surface exhibited a larger WCA of ~154°. However, the average WCA value was reduced slightly at a longer deposition time (12 h). Therefore, the surface deposited for 7 h had an optimum hydrophobic effect. 

**Figure 5:**
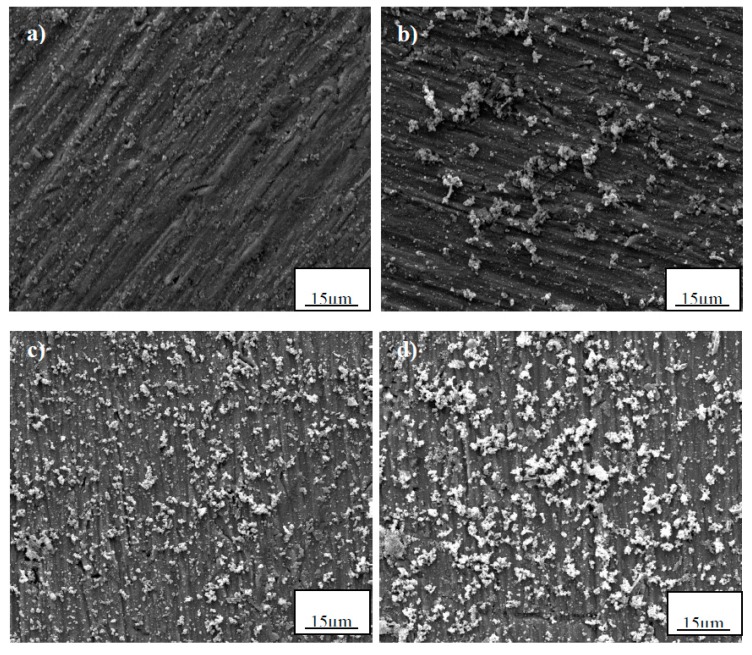
Influence of deposition time on the microstructure of the superhydrophobic surface: (**a**) 0.5 h; (**b**) 2 h; (**c**) 7 h; and (**d**) 12 h.

The SEM morphologies of the hydrophobic surfaces on Ti samples deposited for various times are given in Figure 5. With the increasing deposition time, a larger amount of small, bright particles are deposited and uniformly distributed on the sample surface. As shown in Figure 5c,d, the specimen surface is rather rough and is covered with relatively uniform micro-nano hierarchical structures.

**Figure 6:**
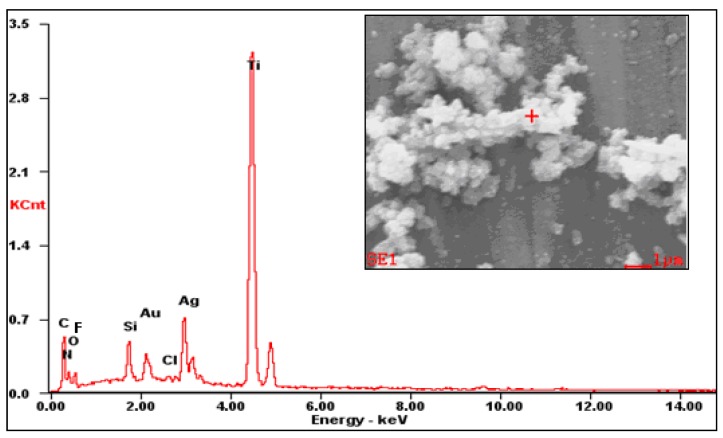
EDS analysis of white packed particles on the superhydrophobic surface.

The composition and morphology of these particles were analyzed by EDS and SEM, respectively, and the results are shown in Figure 6. It consists of a certain content of Ag and other elements. This suggests that the deposited small bright particles are AgNPs, which is attributed to the chelation effect and reducibility of polydopamine (PDA) towards Ag. It is also obvious that AgNPs had agglomerated and formed a micron-sized particulated morphology. Thus, combining with Figure 4 and Figure 5, it is concluded that the Ti surface was covered uniformly by a micro-nano silver structure which induced excellent superhydrophobicity (Figure 5c,d).

**Figure 7:**
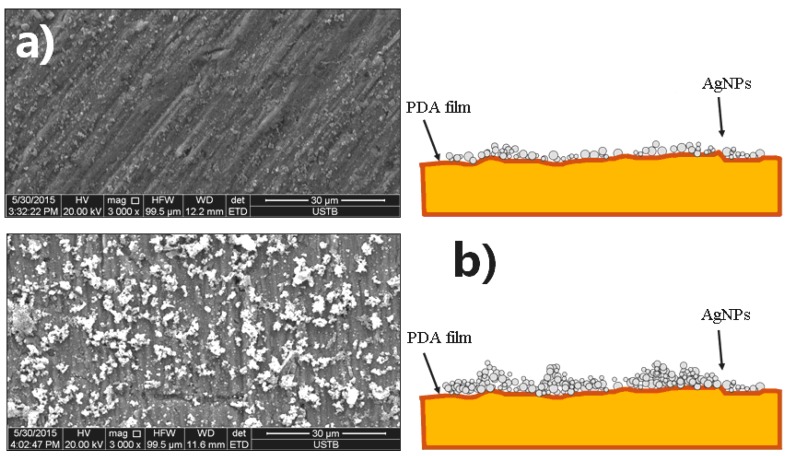
Microstructure of hydrophobic surfaces of specimens prepared at different immersion times in a silver nitrate solution: (**a**) 0.5 h; and (**b**) 12 h.

The above results clearly show that the deposition time plays a key role in the superhydrophobic property of the specimen surface. The main reason is that the deposited quantity of AgNPs increases with the increasing time, which influences the microstructure. When the immersion time was only 0.5 h, a very small amount of AgNPs was deposited on the PDA film and could not effectively constitute the micro-nano stratified microstructure (Figure 7a). In addition, only a small number of thiol bonds could attach to the Ag microstructure during the modification process. Therefore, such a short immersion time could not obtain good hydrophobicity (Figure 4). In contrast, when the deposited time was 12 h, an excess of AgNPs agglomerated together and enhanced the surface roughness to a certain extent (Figure 7b). Thus, an appropriate immersion time is important for constituting the micro-nano structure. 

**Figure 8:**
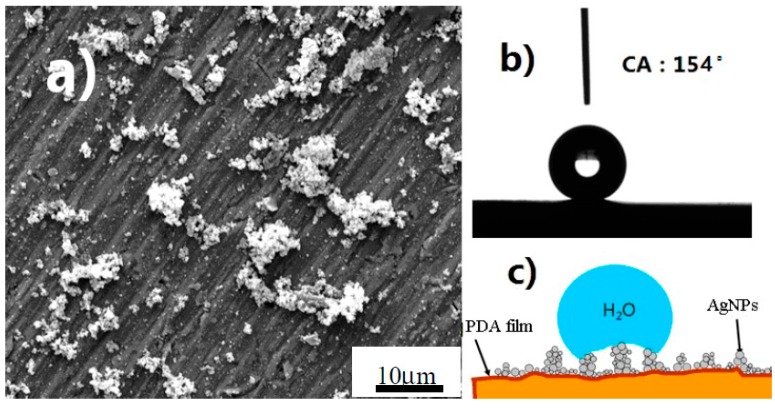
Superhydrophobic surface of specimen prepared at a deposition time of 7 h in a silver nitrate solution: (**a**) microstructure; (**b**) water contact angle; and (**c**) superhydrophobic mechanism.

Figure 8a shows the superhydrophobic surface of specimen prepared at a deposition time of 7 h in silver nitrate solution. The deposition and agglomeration of AgNPs constituted a micron and nanometer structure, which is analogous to the rough microstructure of the lotus leaf. Likewise, the WCA of ~154° indicates that the surface of the specimen exhibits a superhydrophobic characteristic (Figure 8b). In addition, the advancing and receding angles were 156° and 152°, respectively, making the hysteresis 5°. The sliding angle was less than 5°. Figure 8c displays the mechanism schematic of the superhydrophobic surface. Such regularly-ordered microstructures can trap a large amount of air, i.e., forming an air film layer [26]. When water droplets are deposited on the superhydrophobic surface, they can hardly wet the surface of the micron and nanometer hierarchical structure due to the existence of the air film. That is to say, the surface state is accorded with the Cassie mode [7]. 

Thus, by the self-assemblies of the PDA film and AgNPs, a special stratified microstructure was formed on the Ti substrate. After modifying the treatment of 1H,1H,2H,2H-perfluorodecanethiol, the superhydrophobic state can be achieved on the Ti substrate. 

Above all, firstly the polydopamine film layer was prepared on the surface of titanium substrates pretreated by mechanical polishing/anodizing or mechanical polishing only. On the basis of the PDA film, AgNPs were deposited for 7 h using the reductivity of pyrocatechol in dopamine. Through the connection between AgNPs and thiol groups, perfluorinated decanethiol hydrophobic segments were grafted and finally formed a superhydrophobic film structure. The excellent hydrophobic is attributed to the rough micro-nanometer structure along with the low surface energy. Therefore, superhydrophobicity surfaces can be achieved on a titanium substrate with or without anodizing by using the self-assembling method. Thus, the anodizing procedure can be removed, and the fabrication process can be simplified. 

### 3.3. Electrochemical Test 

**Figure 9:**
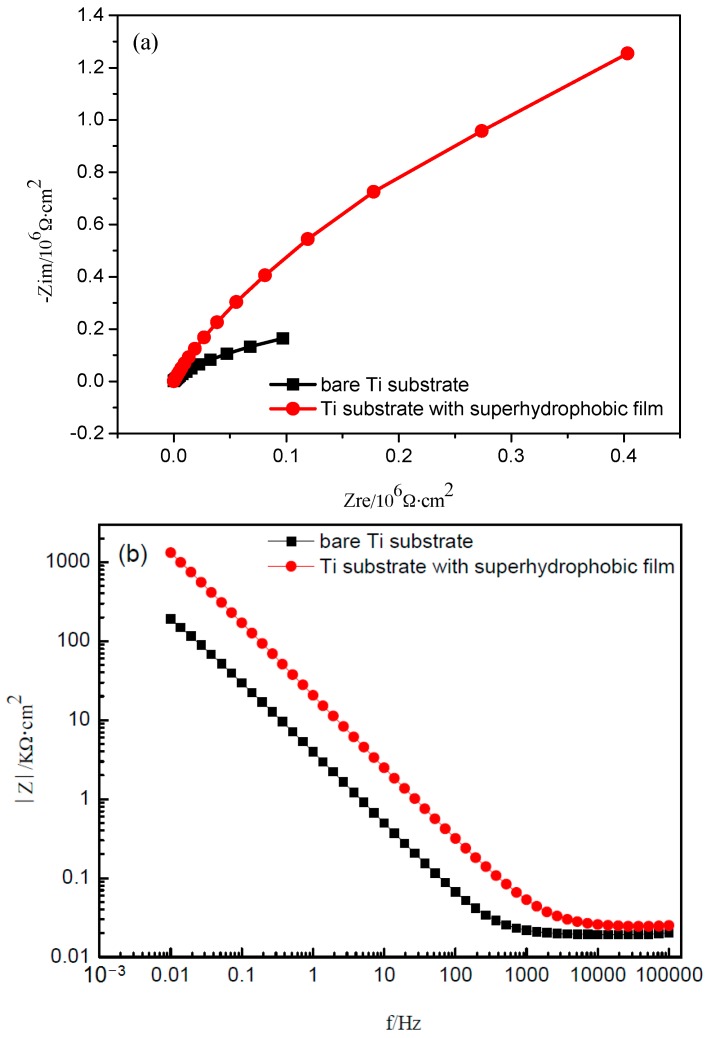
Nyquist diagram (**a**) and Bode diagram (**b**) for the Ti samples with and without the superhydrophobic surface.

Figure 9 displays the impedance spectra for the Ti samples with and without the superhydrophobic surface. As shown in Figure 9a, the curves present a characteristic capacitive arc, and the sample covered by the superhydrophobic film exhibits much larger impedance semicircles whose diameter is around a few thousands of kΩ⋅cm^2^. In contrast, the pure Ti specimen displays a small semicircle. It is generally known that a larger capacitive arc at the low frequency region indicates a better corrosion resistance on the metal substrates [27,28,29]. This suggests that the air layer trapped in the superhydrophobic film is sufficiently continuous. It can effectively prevent the solution medium and dissolved oxygen from contacting the substrate directly [26] and then enhance the anticorrosion property.

Figure 9b shows the Bode plots of Ti samples with and without a superhydrophobic surface. As observed in the plots, the Ti substrate with superhydrophobic film displays a high |Z| value of 1.319 × 10^3^ kΩ⋅cm^2^ at the frequency of 0.01 Hz, which is nearly five times larger than that of the bare Ti substrate at the same frequency. It is well known that the higher |Z| value at the low frequency region signifies a better barrier property of the thin film [30]. Therefore, based on the Bode plots, the superhydrophobic substrate has better corrosion resistance compared with the bare substrate. Again, the excellent anticorrosion property of the superhydrophobicity film is attributed to air trapped in the micro-nano structure, which limits infiltration of water and corrosive species into the interface between the film and substrate [26].

**Figure 10:**
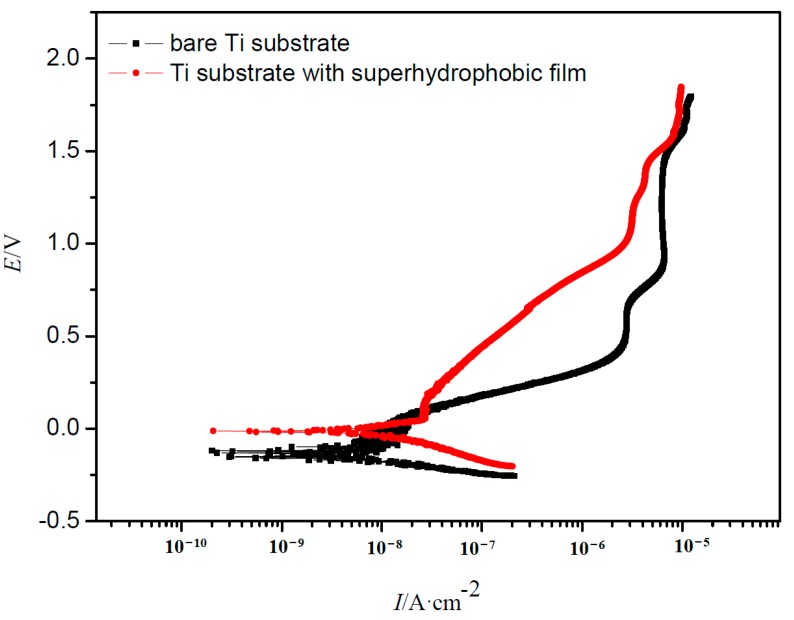
Polarization curves of the Ti samples with and without the superhydrophobic surface.

Figure 10 shows the polarization curves of the Ti samples with and without the superhydrophobic surface. As clearly observed in the graph, the shapes of the curves of the Ti samples with and without the superhydrophobic surface are similar. Both of them displayed obvious passivation characteristics, indicating a stable passive film formed on the sample surface. E_corr_ and I_p_ derived from Figure 10 for different samples are listed in Table 3. The Ti specimen with the superhydrophobic film shows more positive E_corr_ and lower I_p_ as compared with that of the bare Ti substrates. It is generally known that lower corrosion current density values represent lower corrosion dynamic rates, and more positive E_corr_ suggests lower corrosion thermodynamical tendency [31]. This indicates that the corrosion resistance of the sample covered by the superhydrophobic surface is higher than the bare Ti substrate. Thus, the existence of the superhydrophobic film can effectively reduce the anodic dissolution of the Ti specimen [32], and inhibit the occurrence of corrosion. This result is in accordance with that of the EIS test.

## 4. Conclusions 

In this study, superhydrophobic surfaces were prepared on titanium substrate pretreated by mechanical polishing/anodizing or mechanical polishing only. Combined with the self-assemblies of dopamine and AgNPs, and post-modification of 1H,1H,2H,2H-perfluorodecanethiol, superhydrophobic films were obtained on the pretreated sample surfaces. The study also showed that the anodizing procedure can be removed and the fabrication process can be simplified. The hydrophobic property was gradually enhanced with the increasing deposition time in silver nitrate solution. The surface deposited for 7 h exhibited an optimum hydrophobic effect, with a WCA as high as ~154°. The surface was rather rough and covered by relatively uniform micro-nano silver structures. The excellent hydrophobicity was attributed to the rough, stratified microstructure along with the low surface energy. Compared with the bare Ti sample, the specimen with the superhydrophobic film possessed an improved anticorrosion property. 

## Figures and Tables

**Table 1:** Composition of the small white particle surface shown in Figure 2.

Element	CK	NK	OK	FK	AuK	AgK	TiK
wt %	01.15	05.69	02.94	00.50	00.17	15.70	73.85
at %	03.72	15.73	07.11	00.85	00.18	05.09	67.32

**Table 2:** WCA values of surfaces prepared at different immersion times.

Time/h	CA/°			Average CA/°	σ
0.5	147	141	146	144.7	3.2
2	151	150	152	151	1.0
5	152	153	153	152.7	0.6
7	154	154	154	154	0
12	153	152	152	152.3	0.6

**Table 3:** Corrosion potential (E_corr_) and passive current density (I_p_) of the fitted samples.

Samples	Bare Ti	Ti with Film
E_corr_/V(vs. SCE)	−0.135	−0.0126
I_p_/(nA/cm^2^)	8.02	1.22

## References

[1] Zhang X., Shi F., Niu J., Jiang Y., Wang Z. (2008). Superhydrophobic surfaces: From structural control to functional application. J. Mater. Chem..

[2] Roach P., Shirtcliffe N.J., Newton M.I. (2008). Progess in superhydrophobic surface development. Soft Matter.

[3] Satyaprasad A., Jain V., Nema S.K. (2007). Deposition of superhydrophobic nanostructured Teflon-like coating using expanding plasma arc. Appl. Surf. Sci..

[4] Liu K.S., Yao X., Jiang L. (2010). Recent developments in bio-inspired special wettability. Chem. Soc. Rev..

[5] Yan Y.Y., Gao N., Barthlott W. (2011). Mimicking natural superhydrophobic surfaces and grasping the wetting process: A review on recent progress in preparing superhydrophobic surfaces. Adv. Colloid Interface Sci..

[6] Feng L., Li S., Li Y. (2002). Super-hydrophobic surfaces: From natural to artificial. Adv. Mater..

[7] Cassie A.B.D., Baxter S. (1944). Wettability of porous surfaces. Trans. Faraday Soc..

[8] Wenzel R.N. (1936). Resistance of solid surfaces to wetting by water. Ind. Eng. Chem..

[9] Ruan M., Li W., Wang B., Luo Q., Ma F., Yu Z. (2012). Optimal conditions for the preparation of superhydrophobic surfaces on al substrates using a simple etching approach. Appl. Surf. Sci..

[10] Yuan Z., Bin J., Wang X., Peng C., Wang M., Xing S., Xiao J., Zeng J., Xiao X., Fu X. (2014). Fabrication of superhydrophobic surface with hierarchical multi-scale structure on copper foil. Surf. Coat. Technol..

[11] Tsujii K., Yamamoto T., Onda T., Shibuchi S. (1997). Super oil-repellent surfaces. Angew. Chem. Int. Ed. Engl..

[12] Liu Q., Chen D., Kang Z. (2015). One-step electrodeposition process to fabricate corrosion-resistant superhydrophobic surface on magnesium alloy. ACS Appl. Mater. Interfaces.

[13] Guo X., Li X. (2017). An expanding horizon: Facile fabrication of highly superhydrophobic coatings. Mater. Lett..

[14] Latthe S.S., Imai H., Ganesan V., Rao A.V. (2009). Superhydrophobic silica films by sol–gel co-precursor method. Appl. Surf. Sci..

[15] Zhang D.W., Wang L.T., Qian H.C., Li X.G. (2016). Superhydrophobic surfaces for corrosion protection: A review of recent progresses and future directions. J. Coat. Technol. Res..

[16] He T., Wang Y.C., Zhang Y.J., Iv Q., Xu T.G., Liu T. (2009). Super-hydrophobic surface treatment as corrosion protection for aluminum in seawater. Corros. Sci..

[17] Xu W., Song J., Sun J., Lu Y., Yu Z. (2011). Rapid fabrication of large-area, corrosion-resistant superhydrophobic Mg alloy surfaces. ACS Appl. Mater. Interfaces.

[18] Liu T., Chen S., Cheng S., Tian J., Chang X., Yin Y. (2007). Corrosion behavior of super-hydrophobic surface on copper in seawater. Electrochim. Acta.

[19] Liu H., Szunerits S., Xu W., Boukherroub R. (2009). Preparation of superhydrophobic coatings on zinc as effective corrosion barriers. ACS Appl. Mater. Interfaces.

[20] Zhang D.W., Qian H.C., Wang L.T., Li X.G. (2016). Comparison of barrier properties for a superhydrophobic epoxy coating under different simulated corrosion environments. Corros. Sci..

[21] Han I., Vagaska B., Seo H.J., Kang J.K., Kwon B.J., Lee M.H., Park J.C. (2012). Promoted cell and material interaction on atmospheric pressure plasma treated titanium. Appl. Surf. Sci..

[22] Joung Y.S., Buie C.R. (2013). A hybrid method employing breakdown anodization and electrophoretic deposition for superhydrophilic surfaces. J. Phys. Chem. B.

[23] Lu Y., Xu W.J., Song J.L., Liu X., Xing Y.J., Sun J. (2012). Preparation of superhydrophobic titanium surfaces via electrochemical etching and fluorosilane modification. Appl. Surf. Sci..

[24] Vanithakumari S.C., George R.P., Mudali U.K. (2013). Enhancement of corrosion performance of titanium by micro-nano texturing. Corrosion.

[25] Liang J.S., Liu K.Y., Wang D.Z. (2015). Hollow organosilica nanospheres prepared through surface hydrophobic layer protected selective etching. Appl. Surf. Sci..

[26] Doshi D.A., Shah P.B., Singh S., Branson E.D., Malanoski A.P., Watkins E.B., Majewski J., Swol V.F., Brinker C.J. (2005). Investigating the interface of superhydrophobic surfaces in contact with water. Langmuir.

[27] Vreugdenhil A.J., Gelling V.J., Woods M.E., Schmelz J.R., Enderson B.P. (2008). The role of crosslinkers in epoxy–amine crosslinked silicon sol–gel barrier protection coatings. Thin Solid Films.

[28] Qian M., Mcintosh A.S., Tan X.H., Zeng X.T., Wijesinghe S.L. (2009). Two-part epoxy-siloxane hybrid corrosion protection coatings for carbon steel. Thin Solid Films.

[29] Xue C.R., Dong L.H., Liu T., Zhang F., Yin B., Yin Y.S. (2012). Preparation and anticorrosion performance of superhydrophobic TiO_2_ nanotube arrays on pure Ti. Corros. Sci. Prot. Technol..

[30] Zhang F., Chen S., Dong L., Lei Y., Liu T., Yin Y. (2011). Preparation of superhydrophobic films on titanium as effective corrosion barriers. Appl. Surf. Sci..

[31] Ou J.F., Liu M.Z., Li W., Wang F.J., Xue M.S., Li C.Q. (2012). Corrosion behavior of superhydrophobic surfaces of Ti alloys in NaCl solutions. Appl. Surf. Sci..

[32] Sagert J., Sun C., Waite J.H., Smith A.M., Callow J.A. (2006). Chemical subtleties of Mussel and Polychaete holdfasts. Biological Adhesives.

